# Can artificial intelligence help for scientific illustration? Details matter

**DOI:** 10.1186/s13054-024-04970-8

**Published:** 2024-06-10

**Authors:** Julian Klug, Urs Pietsch

**Affiliations:** 1 Division of Perioperative Intensive Care Medicine, Cantonal Hospital St. Gallen, Rorschacher Strasse 95, 9007 St. Gallen, Switzerland; 2 Stroke Research Group, Department of Clinical Neurosciences, Faculty of Medicine, University Hospital, Geneva, Switzerland; 3 Department of Emergency Medicine, Inselspital, Bern University Hospital, University of Bern, Bern, Switzerland

Over the past years, there has been a growing use of generative artificial intelligence (AI) in scientific publications, including for the creation of scientific illustrations. Generative AI refers to a subset of AI models that focus on the creation or modification of content such as images or text. The images generated are of high quality and effectively convey complex concepts [1]. Aesthetically pleasing at first glance, singular details may reveal that an image has been created by AI. Most commentaries on generative AI in the scope of scientific publishing focus on the generation of text, including a recent perspective in this journal [2]. The use of image generation tools for scientific illustration has generated less interest and remains mostly unregulated [3]. The aim of this work is to present an overview of the current AI image generation tools for scientific illustration, including an analysis of their potential benefits and associated risks.

In practice, generative text-to-image models take as input a “prompt”, a short piece of descriptive text, and proceed to generate a matching image. The models are trained on text-image pairs and are made from two main components: an encoder that generates an image embedding given a text caption, and a decoder diffusion model that generates an image conditioned on the image embedding. The most commonly used models include DALL-E (OpenAI), MidJourney (Midjourney, Inc.) and Stable Diffusion (Stability AI). Although researchers and medical professionals have a common knowledge base in the usage of the written word, illustration and manipulation of complex image-editing software are rarely taught in the current academic syllabus. AI image generation tools can help create both clinical images and conceptual illustrations, as well as image parts or icons. Providing an interactive platform for doodling, these tools can speed up the iterative creative process, generating a first draft in seconds. On a basic level, most tools contain an interactive text interface from which a prompt can be entered and modified, with an image being generated accordingly. More advanced techniques include the generation of variations of an existing image, creating an image from an existing sketch, completing around, or filling in areas of an existing image as well as removing parts of an image (Table 1).

**Table 1:** Overview of commonly used AI image-generation techniques

Technique	Explanation
Text-to-image generation	Generation of a de novo image given a text prompt
Image variation	Generation of variations of an existing image
Sketch-to-image	Generation of an image given a sketch and a text prompt
Object removal	Removal of parts of an existing image
In-painting	Generation of new content inside a marked area of an existing image given a text prompt
Out-painting	Generation of new content around an existing image given a text prompt

However, the use of AI tools for scientific illustration is not without pitfalls. A highly publicized example was a now retracted article, in which AI was used to create images of the JAK-STAT signaling pathway and the rodent reproductive system that were entirely fictional [4]. Interestingly, the work had passed peer-review. This example highlights the shortcomings of AI tools in scientific illustration, producing inaccurate, physically impossible, and misleading images whilst maintaining a falsely scientific aesthetic. Such grotesque deformation of reality would fool no reader, but a more subtle misrepresentation could go unnoticed [5]. This can be particularly problematic in some open-access journals with expedited editorial and review processes. Malicious intent aside, as AI generated images are usually of high graphical quality and often visually stunning, inaccuracies can creep in and be overlooked at first glance by authors and reviewers alike. For example, when evaluated on the creation of anatomical images, text-to-image tools fail to render important landmarks such as the foramina and sutures of the human skull [6]. Other typical misrepresentations in generated images are text labels consisting of letter-like symbols and abnormal branching of linear structures such as arteries or veins [7]. As in other domains of human–machine interaction, such as image analysis [8], special attention is required to avoid automation bias, in which users might be prone to more readily accept a model’s output and/or distance themselves from the results, losing accountability. Further, as with AI generated text, the intended tone is often slightly deformed and generated images with medical content tend to appear slightly futuristic and gruesome in nature. This is likely due to the training data, which is sourced from the internet and thus contains large amounts of fictional content [9].

Several ethical concerns arise about the use of generative AI for scientific illustration. Generative image models have been shown to be able to reproduce training samples at inference ranging from photographs of individual people to trademarked images [10]. Consequently, these systems can commit overt plagiarism without the user’s knowledge and the consent for the use of the original photos for the creation of the model remains problematic. Conversely, publishing traditional patient photos is limited by concerns over confidentiality, especially when facial features are essential. Here text-to-image AI tools may offer an alternative by producing accurate images without disclosing identifiable patient information [11]. However, being trained on the current distribution of available imagery, generative AI tools pose a risk to perpetuate and amplify existing biases and stereotypes of oversimplified social categorization. For example, when prompted to generate images of surgeons, over 98% are depicted as White and male [12]. Contrarily, when instructed to create an image of suffering children, the patients are unvaryingly depicted as Black [13]. Users of generative AI tools therefore must be conscious of the inherent biases to construct prompts that generate representations that accurately depict the demographic realities of our society.

There is no doubt that AI image generation will significantly influence scientific communication. Whether it will be marked by improved illustrations of educational potential or misleading distortions of reality is yet to be determined. As with the ongoing debate on AI-generated text, the scientific community should engage in an open discourse regarding their use to establish best practices for authors and reviewers alike. In this context, we would like to highlight the recently published corresponding guidelines by the European Commission, stating that authors should remain ultimately responsible for the scientific output and be transparent about the use of AI tools [14]. Individual publishers have come forward with their own regulations, and not all permit the use of generative AI [15]. When allowed, the use of generative AI to create images should be stated openly. The conceptual nature of the generated images must be explicit and immediately clear to readers, avoiding the possibility of misinterpretation.

Practically speaking, generative AI represents an appealing tool to simplify, speed up and lower costs of scientific illustration, but active critical appraisal is needed to ensure minimal standards are met. Inaccurate representations should not be allowed to pass the editorial and peer review. As with clinical monitoring, practitioners cannot blindly rely on the output of a single model. Fortunately, by using multiple models sequentially, inaccuracies can be easily corrected using techniques such as object removal and inpainting, as demonstrated in Fig. 1. Finally, sometimes the authors must resort to traditional image editing software to correct remaining imperfections.

**Fig. 1:**
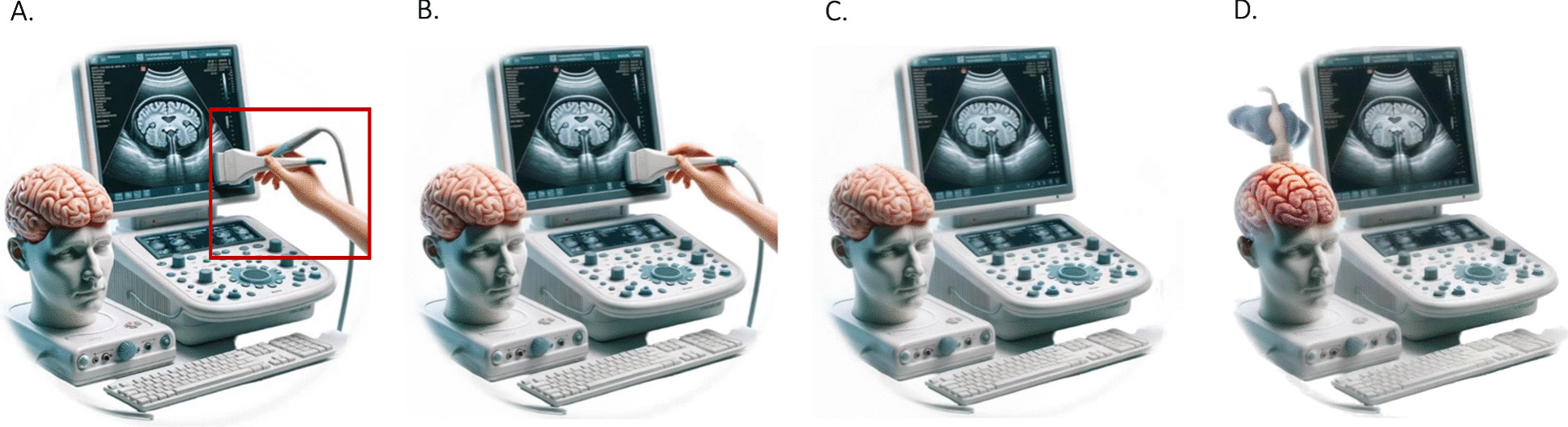
Example of iterative image correction using generative AI. Demonstration of transcranial ultrasound on a phantom. **A** Selection of the part of the image to be corrected. **B** Correction of inaccuracies by inpainting with prompt “cable joining end of probe being held in hand”. **C** Removal of inaccuracies by automated object removal. **D** End result after multiple iterations. Stable diffusion was used as text-to-image model for all computations

Easy to access and integrated into commonly used tools, AI is starting to penetrate every stage of scientific publishing. We believe that generative text-to-image models can simplify and enhance the creation of scientific illustrations. However, we have noticed that their use can sometimes lead to a loss of attention to detail. We therefore encourage authors to (1) declare the use of AI tools, (2) critically scrutinize and iteratively correct any inaccuracies arising during the generative process and (3) ensure generated images are free of bias. We should remember the words of the well-known technology pioneer, Steve Jobs: "Details matter. It's worth waiting to get it right."

## Data Availability

Not applicable.

## Abbreviations

AI: Artificial intelligence
